# Abnormal Amygdala Subregion Functional Connectivity in Patients with Crohn's Disease with or without Anxiety and Depression

**DOI:** 10.1155/2024/1551807

**Published:** 2024-01-30

**Authors:** Jingwen Sun, Wei Sun, Kecen Yue, Yin Zhang, Xintong Wu, Wenjia Liu, Ling Zou, Haifeng Shi

**Affiliations:** ^1^Department of Radiology, The Affiliated Changzhou No. 2 People's Hospital of Nanjing Medical University, Changzhou, China; ^2^Graduate College, Dalian Medical University, Dalian, China; ^3^School of Computer and Artificial Intelligence, Changzhou University, Changzhou, China; ^4^Department of Gastroenterology, The Affiliated Changzhou No. 2 People's Hospital of Nanjing Medical University, Changzhou, China

## Abstract

**Objective:**

The aim of this study was to explore the resting-state functional connectivity (rsFC) of amygdala subregions in healthy controls (HCs) and in patients with Crohn's disease (CD) both with and without anxiety or depression.

**Materials and Methods:**

A total of 33 patients with CD and with anxiety or depression (CDad group), 31 patients with CD but without anxiety or depression (CDnad group), and 29 age-, sex-, and education level-matched HCs underwent functional magnetic resonance imaging. rsFC analysis was used to analyze the FC between the amygdala subregions and other areas of the brain.

**Results:**

Compared with the HC group, the CDad group demonstrated decreased rsFC between the right laterobasal subregion and the left hippocampus (*P* < .001) and right middle frontal gyrus (*P* < .001) and between the left superficial subregion and the left insula (*P* < .001). Compared with the HC group, the CDnad group demonstrated decreased rsFC between the left centromedial subregion and the left insula (*P* < .001). Compared with the CDnad group, the CDad group demonstrated decreased rsFC between the left centromedial subregion and the right precuneus (*P* < .001) and postcentral gyrus (*P* < .001), between the right laterobasal subregion and the left hippocampus (*P* < .001), and between the left superficial subregion and the right middle frontal gyrus (*P* < .001).

**Conclusions:**

There are significant FC changes in the amygdala subregions in patients with CD. These changes may be related to the disease itself or to the symptoms of anxiety and depression.

## 1. Introduction

Crohn's disease (CD) is a chronic inflammatory disorder that affects the gastrointestinal tract and has a substantial impact on quality of life. Recent studies have suggested that stress and mood disorders can adversely affect the course of CD via a mechanism that has not yet been fully elucidated [1, 2].

One potential mechanism underlying the adverse effects of mood disorders on CD involves the amygdala. The amygdala, a component of the limbic system, plays an important role in many psychiatric disorders such as major depressive disorder and posttraumatic stress disorder [3], and overactivation of the amygdala has been identified as an important trigger for anxiety disorders [4]. The amygdala is a heterogeneous nucleus that can be divided into 3 major areas based on cytoarchitectonic characteristics: the laterobasal (LB), centromedial (CM), and superficial (SF) subregions [5]. The CM subregion is linked to brain regions involved in motor function, perception, and processing of somatosensory and visceral inputs [6]. The LB subregion is connected to earlier visual cortices and several sensory areas responsible for processing higher-level visual and auditory inputs [6]. The SF subregion is linked to brain regions involved in reward prediction, olfaction, and affective and vegetative processing [6].

Structural and functional abnormalities in the amygdala have been demonstrated in multiple magnetic resonance imaging (MRI) studies in patients with CD [7, 8]. For instance, Rubio et al. [9] found that amygdala activation was significantly stronger in patients with CD than in control participants, and Bao et al. [10] found that amygdala volume was increased in patients with CD. These studies assessed alterations of the amygdala, but the potential role of amygdala subregions in CD has not been fully investigated. In addition, although extensive research has been conducted regarding brain function in patients with CD who suffer from anxiety or depression [11], limited studies have been dedicated to investigating brain function in patients with CD who do not experience anxiety or depression.

Resting-state functional connectivity (rsFC) can be evaluated with functional MRI (fMRI), which is defined as the correlation of activity between brain regions that are spatially distant [12]. Positive blood oxygen level-dependent (BOLD) signals between brain regions indicate functional synergies, whereas negatively correlated signals indicate functional antagonism. By identifying the interaction between BOLD signals from different brain regions, FC imaging can be used to detect abnormalities in brain function and to help diagnose and evaluate diseases in various patient populations. The functional connectivity alterations within the subregions of the amygdala have been extensively studied in many ailments [12, 13]. However, to my understanding, no exploration has been conducted on Crohn's disease.

The goal of this study, therefore, was to identify abnormalities in rsFC in the amygdala subregions in patients with CD both with and without symptoms of anxiety or depression and in healthy control (HC) participants to determine whether these abnormalities were associated with the CD itself or with the mood disorder symptoms.

## 2. Materials and Methods

### 2.1. Participants

This prospective study followed the principles outlined in the Declaration of Helsinki. Approval was obtained from the Ethics Committee of the Affiliated Changzhou No. 2 People's Hospital of Nanjing Medical University (KY026-01), and all study participants provided written informed consent.

Patients who were eligible for inclusion in the CD group were as follows: (1) aged 18 to 70 years, were right-handed, (2) had an endoscopic and/or histological diagnosis confirmed by skilled endoscopists at the hospital, and (3) had a disease that had been in remission for at least 6 months. The inclusion criteria of the healthy control group were as follows: (1) 18 to 70 years, were right-handed, and (2) had no history of any bowel disease. Participants (both patients with CD and HCs) who were excluded from analysis were as follows: (1) if they had used corticosteroids or psychotropic drugs in the previous 90 days; (2) if they had a current or past history of neurological or mental illness; (3) if structural MR images showed organic abnormalities of the head; (4) if they had a current or past history of neurosurgery, head injury, cerebrovascular injury, or brain trauma involving loss of consciousness; and (5) if they had magnetic implants anywhere in the body.

Of 77 patients who were initially considered for study inclusion, 70 were enrolled in the study. However, 4 patients had partial image loss during data preprocessing, and 2 patients were excluded because of excessive head movement. Therefore, the final CD group consisted of 64 patients. Additionally, 29 healthy patients who underwent physical examination at the same hospital and were similar to the CD group in age, sex, and education levels were included in the control group. Data on sex, age, height, weight, and education level were collected from all study participants.

### 2.2. Questionnaires

Depression and anxiety levels were assessed using the Hospital Anxiety and Depression Scale (HADS). Scores of 0 to 7 were considered normal, whereas scores of 11 or higher indicated a probable mood disorder. Scores of 8 to 10 were considered borderline. Based on these scores, patients with CD were divided into those with anxiety/depression (CDad group; scores ≥ 8) and those without anxiety or depression (CDnad group; scores ≤ 7).

### 2.3. MRI Data Acquisition

For all participants, resting-state functional images were collected using an 8-channel head coil on a 3.0 T MRI scanner (Achieva, Philips, the Netherlands). Participants were instructed to keep their eyes closed, remain still and alert, and avoid thinking or recalling specific events. High-resolution T1 structural images were obtained using the following parameters: repetition time, 8.2 ms; echo time, 3.7 ms; field of view, 256 × 256 mm^2^; flip angle, 9°; matrix size, 256 × 256; slice thickness, 1 mm; and voxel size, 1 × 1 × 1 mm^3^; and the total scan time was 7 min 6 s. A whole brain gradient echo planar sequence was used with the following parameters: repetition time, 2000 ms; echo time, 30 ms; field of view, 192 × 192 mm^2^; flip angle, 90°; matrix size, 64 × 64; slice thickness, 3 mm; voxel size, 3 × 3 mm^2^; and time points, 240. The total scan time was 7 min 6 s.

### 2.4. MRI Data Preprocessing

Preprocessing was performed using the SPM12 toolbox (SPM12 Software—Statistical Parametric Mapping (https://ucl.ac.uk). Participant scans with poor image quality were excluded. The fMRI data were corrected for head movement, registered with T1 data, and then we segmented T1 data into gray matter, white matter, and cerebrospinal fluid by DARTEL, spatial standardized by the probability map of gray matter after segmentation. Then, the data were resampled (2 × 2 × 2 mm^3^) and smoothed with a full width at half-maximum kernel of 4 mm. A regression analysis was performed on Friston head motion parameters. The data were also filtered between .01 and .08 Hz.

### 2.5. Definition of Regions of Interest

**Figure 1:**
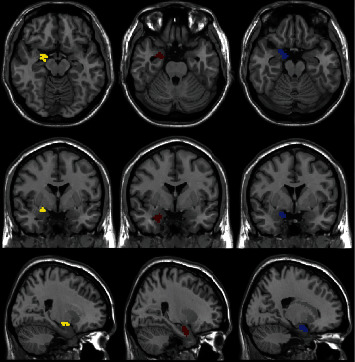
Images demonstrating amygdala subregions on the left side of the brain. The centromedial subregion is indicated with yellow, the basolateral subregion is indicated with red, and the superficial subregion is indicated with blue.

SPM anatomy software (http://www.fz-juelich.de/inm/inm-1/DE/Forschung/_docs/SPM Anatomy Toolbox/SPMAnatomyToolbox_node.html) was used to acquire templates for the CM, BL, and SF nuclei of the amygdala (Figure 1). Probabilistic maps from the JuBrain Cytoarchitectonic Atlas were employed to create region of interest masks based on the cytoarchitecture of the amygdala [5].

### 2.6. rsFC Analysis

rsFC analysis has been performed utilizing DPARSF. The time series from each voxel within target regions were extracted. Subsequently, we calculated the mean time series by averaging the time series of each voxel inside target regions. Then, the rsFC maps between each subregion of the amygdala and the whole brain were then computed as the Pearson correlation coefficient for each participant. The correlation coefficients were then transformed into *z* values using Fisher's *r*-to-*z* transform to enhance normality. This resulted in 6 *z*-score maps for each participant, representing the intrinsic rsFC patterns of the 6 amygdala subdivisions.

### 2.7. Statistical Analysis

For continuous variables, a one-way analysis of variance (ANOVA) was used; for categorical variables, a chi-square test was used. FC differences among the 3 groups were identified using ANOVA, and 2-sample *t*-tests were used to compare pairs of groups. The significance thresholds were set at *P* < .001 for voxel-level and *P* < .05 for cluster-level family-wise error rate corrections, and the minimum cluster was eight voxels.

## 3. Results

### 3.1. Clinical and Demographic Characteristics

Table 1 displays the clinical and demographic characteristics of the study participants. No significant differences in sex, age, height, weight, or education level were observed among the groups. The CDad group had higher anxiety and depression scores than the CDnad group and the HC group (*P* < .001).

### 3.2. Results of rsFC Analysis

**Figure 2:**
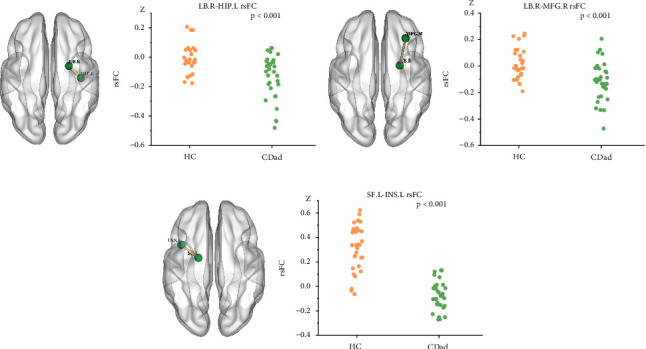
Healthy control (HC) participants and patients with Crohn's disease and with anxiety or depression (CDad) demonstrated significant differences in resting-state functional connectivity (rsFC) across various brain regions. (a-c) Outcomes of reduced functional connectivity, with each set of images comprising a left-sided brain region and a corresponding scatter plot on the right. LB = laterobasal subregion; SF = superficial subregion; HIP = hippocampus; MFG = middle frontal gyrus; INS = insula.

Compared with the HC group, the CDad group demonstrated decreased rsFC between the right LB and the left hippocampus (*P* < .001, *k* ≥ 8 voxels) and right middle frontal gyrus (MFG) (*P* < .001, *k* ≥ 8 voxels). The CDad group also demonstrated decreased rsFC between the left SF and the left insula (*P* < .001, *k* ≥ 8 voxels) (Figure 2).

**Figure 3:**
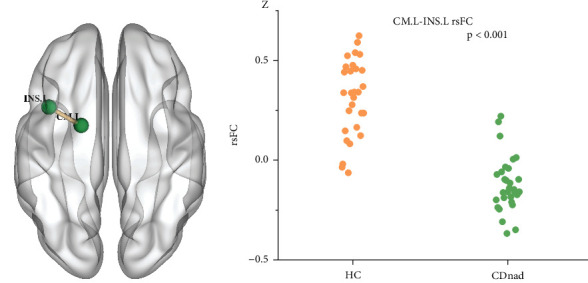
Healthy control (HC) participants and patients with Crohn's disease but without anxiety or depression (CDnad) demonstrated significant differences in resting-state functional connectivity (rsFC) across various brain regions. (a) Brain regions demonstrating significant differences in rsFC. (b) Scatter plots of mean rsFC values in the 2 groups. CM = centromedial subregion; INS = insula.

Compared with the HC group, the CDnad group demonstrated decreased rsFC between the left CM and the left insula (*P* < .001, *k* ≥ 8 voxels) (Figure 3).

**Figure 4:**
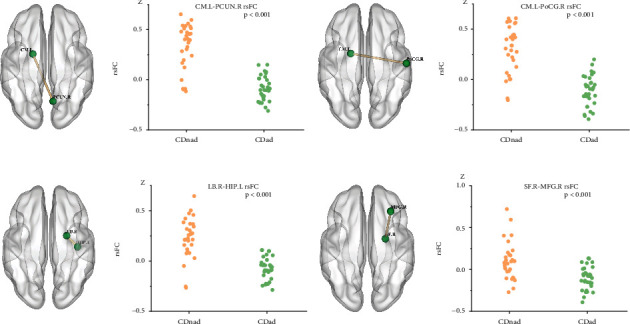
Patients with Crohn's disease and with anxiety or depression (CDad) and patients with Crohn's disease but without anxiety or depression (CDnad) demonstrated significant differences in resting-state functional connectivity (rsFC) across various brain regions. (a-d) The outcomes of reduced functional connectivity, with each set of images comprising a left-sided brain region and a corresponding scatter plot on the right. CM = centromedial subregion; LB = laterobasal subregion; SF = superficial subregion; PCUN = precuneus; PoCG = postcentral gyrus; HIP = hippocampus; MFG = middle frontal gyrus.

Compared with the CDnad group, the CDad group demonstrated decreased rsFC in many regions, including between the left CM and the right precuneus (*P* < .001, *k* ≥ 8 voxels) and right postcentral gyrus (*P* < .001, *k* ≥ 8 voxels), between the right LB and the left hippocampus (*P* < .001, *k* ≥ 8 voxels), and between the SF and the right MFG (*P* < .001, *k* ≥ 8 voxels) (Figure 4).

## 4. Discussion

In this study, we explored the intrinsic FC of amygdala subregions in HC participants and in patients with CD, both with and without symptoms of anxiety or depression. We found significant differences across brain regions in pairwise comparisons of all 3 groups. Our findings suggest that there are significant FC changes in the amygdala subregions among patients with CD and that these changes may be related either to the disease itself or to the accompanying symptoms of anxiety and depression.

First, we found that the CDad group, when compared with the HC group, had decreased rsFC between the right LB and the left hippocampus. The hippocampus is a brain region that is highly sensitive to stress and plays a crucial role in regulating emotions, cognition, memory, and other neural and endocrine pathways [13]. Abnormal function of the hippocampus can lead to various symptoms of depression, with previous studies showing a correlation between major depressive disorder and smaller hippocampal volumes [14]. The hippocampus also plays a major role in regulating the hypothalamus-pituitary-adrenal (HPA) axis, which is crucial for stress response and is also part of the brain-gut axis. Research has shown that the composition of gut microbiota can impact behaviors associated with depression and anxiety in mice. Glucocorticoid receptor pathway genes were found downregulated in the hippocampus of mice with anxiety- and depressive-like phenotypes [15]. This indicated that the gut microbiota may lead to behavioral abnormalities in mice through the glucocorticoid receptor pathway in the hippocampus. Our findings suggest that alterations in the hippocampus could be attributed to both the CD itself and to the associated mood disorder symptoms.

Decreased rsFC was also seen between the right LB and the right MFG in the CDad group compared with the HC group. The MFG, an important component of the medial prefrontal cortex, is involved in cognitive control functions and negative cognition bias [12, 16]. Emotional regulation in the amygdala is controlled by top-down inhibition of the medial prefrontal cortex [17]. Decreased inhibitory control by the MFG on the LB may therefore increase emotional dysfunction [12], which could have contributed to the alterations in MFG FC observed in the CDad group in this study.

Next, we found that the CDnad group demonstrated decreased rsFC between the left CM and the left insula compared with the HC group. The insula is involved in a vast array of functions, including sensory processing, emotion representation, risk assessment and decision-making, self-awareness, and complex social behaviors [18–22]. In addition, a recent study found that the activity of insular cortex neurons increased during the induction of colitis and that inhibiting the activity of these neurons weakened the cellular immune response in the colon [23]. These findings suggest that the changes in insula function are independent of anxiety and depression symptoms and are caused by CD itself, results confirmed by the current study findings.

Finally, we found that in comparison with the CDnad group, the CDad group demonstrated decreased rsFC across many brain regions, including between the left CM and the right precuneus and right postcentral gyrus, between the right LB and the left hippocampus, and between the SF and the right MFG. Located on the medial side of the brain, the precuneus has received relatively little attention in neuroimaging but is thought to be related to the spatial aspects of motor function [24]. One study demonstrated that the precuneus has high FC with the medial prefrontal cortex and hippocampus [25]. The decreased FC in MFG and hippocampus seen in this study may explain the damage to the precuneus. The postcentral gyrus, especially the somatosensory cortex, is responsible for processing sensory information from various parts of the body. The somatosensory cortex also plays a role in emotion recognition; participants with damage to the somatosensory cortex are impaired in their ability to assess the intensity of emotions [26, 27]. One potential mechanism of the emotion recognition ability in the somatosensory cortex could be through the connection of the postcentral gyrus to the amygdala [26, 27]. As a central area involved in emotional regulation, the postcentral gyrus may serve as an important focus of therapy for mood disorders seen extensively in CD. Our findings in the comparison between the CDad group and the CDnad group suggest that these brain regions with decreased FC in the CDad group are more associated with mood disorders rather than CD itself.

In addition, decreased FC in the insula, the hippocampus, and the MFG appeared in multiple results of comparison. The result in comparison between the CDnad group and the HC group could potentially account for the notable difference in the insula observed in the comparison between the HC group and the CDad group, that is the decreased FC in the insula is mainly attributed to CD itself. Similarly, in comparison with the HC group, the observed decreased FC in the MFG and hippocampus in the CDad group may be primarily attributable to mood disorders.

There are some limitations in this study. The division of amygdala subregions was based on the cellular probability map of Amunts et al. [5]; however, it has been suggested that the location of amygdala subregions may be highly variable in each individual [28]. Additionally, cross-sectional studies limit our exploration of other factors that affect the disease, such as the patient's disease duration and treatment options, and further cohort studies are needed.

## 5. Conclusion

In this study, we were able to use rsFC assessment of amygdala subregions to distinguish between brain regions affected by CD itself and those affected by the symptoms of anxiety and depression. This study provides new perspectives and further evidence for the pathophysiology of CD by revealing these changes in brain connectivity.

## Figures and Tables

**Table 1:** Clinical and demographic characteristics of study participants.

Characteristic	CDad group (*n* = 33)	CDnad group (*n* = 31)	HC group (*n* = 29)	*P* value
No. of men/women	18/15	13/18	15/14	.605^b^
Age, years	29.53 ± 7.08	27.66 ± 8.20	30.49 ± 5.84	.413^a^
Height (cm)	169.37 ± 6.91	170.23 ± 5.69	170.92 ± 6.70	.397^a^
Weight (kg)	58.90 ± 7.53	60.90 ± 5.24	60.17 ± 8.22	.548^a^
HADS anxiety score	6.61 ± 1.523	4.79 ± 1.197	4.50 ± 1.122	<.001^a^
HADS depression score	6.86 ± 1.627	4.78 ± 1.258	4.66 ± 1.278	<.001^a^
Education, years	12.85 ± 3.87	14.13 ± 4.39	12.90 ± 4.50	.216^a^

Unless otherwise indicated, data are mean ± SD. CDad = patients with Crohn's disease and with anxiety or depression; CDnad = patients with Crohn's disease but without anxiety or depression; HC = healthy control; HADS = Hospital Anxiety and Depression Scale. ^a^One-way ANOVA. ^b^Chi-square test.

## Data Availability

The data that support the findings of this study are available from the corresponding authors upon reasonable request.
